# De Novo Transcriptome Sequencing Analysis and Comparison of Differentially Expressed Genes (DEGs) in *Macrobrachium rosenbergii* in China

**DOI:** 10.1371/journal.pone.0109656

**Published:** 2014-10-20

**Authors:** Hai Nguyen Thanh, Liangjie Zhao, Qigen Liu

**Affiliations:** 1 Key Laboratory of Freshwater Fishery Germplasm Resources, Shanghai Ocean University, Ministry of Agriculture, Shanghai City, P. R. China; 2 Vietnam Institute of Fisheries Economics and Planning, Directorate of Fisheries, Ministry of Agriculture and Rural Development of Viet Nam, Hanoi City, S.R. Vietnam; Federal University of Rio de Janeiro, Brazil

## Abstract

Giant freshwater prawn (GFP; *Macrobrachium rosenbergii*) is an exotic species that was introduced into China in 1976 and thereafter it became a major species in freshwater aquaculture. However the gene discovery in this species has been limited to small-scale data collection in China. We used the next generation sequencing technology for the experiment; the transcriptome was sequenced of samples of hepatopancreas organ in individuals from 4 GFP groups (A1, A2, B1 and B2). De novo transcriptome sequencing generated 66,953 isogenes. Using BLASTX to search the Non-redundant (NR), Search Tool for the Retrieval of Interacting Genes (STRING), and Kyoto Encyclopedia of Genes and Genome (KEGG) databases; 21,224 unigenes were annotated, 9,552 matched unigenes with the Gene Ontology (GO) classification; 5,782 matched unigenes in 25 categories of Clusters of Orthologous Groups of proteins (COG) and 20,859 unigenes were consequently assigned to 312 KEGG pathways. Between the A and B groups 147 differentially expressed genes (DEGs) were identified; between the A1 and A2 groups 6,860 DEGs were identified and between the B1 and B2 groups 5,229 DEGs were identified. After enrichment, the A and B groups identified 38 DEGs, but none of them were significantly enriched. The A1 and A2 groups identified 21,856 DEGs in three main categories based on functional groups: biological process, cellular_component and molecular function and the KEGG pathway defined 2,459 genes had a KEGG Ortholog - ID (KO-ID) and could be categorized into 251 pathways, of those, 9 pathways were significantly enriched. The B1 and B2 groups identified 5,940 DEGs in three main categories based on functional groups: biological process, cellular_component and molecular function, and the KEGG pathway defined 1,543 genes had a KO-ID and could be categorized into 240 pathways, of those, 2 pathways were significantly enriched. We investigated 99 queries (GO) which related to growth of GFP in 4 groups. After enrichment we identified 23 DEGs and 1 KEGG PATHWAY ‘ko04711’ relation with GFP growth.

## Introduction

The GFP is one of the two most popularly cultured freshwater species in China that belong to genus *Macrobrachium*. Recent reports indicate that average world annual GFP production has surpassed 500,000 tons annually with a value of US$ 2.5 billion and that the culture industry for GFP now exceeds US$ 1.4 billion per year in Asia alone [1]–[3]. As a consequence, there is growing interest in GFP culture, particularly in Asia [4], [5]. To increase productivity of farmed GFP there is a need to better understand the basic biology, ecology and production traits of this species to allow development of more productive culture strains for the expanding global industry.

China is a country with no natural distribution of GFP, thus it has had to be introduced from tropical and subtropical countries since 1976 [6]. Due to its high value, researchers now focus on improving the growth performance of farmed GFP [4], [7]–[10]. It is now the major species in aquaculture and the culture areas have expanded continuously for more than 3 decades. The total GFP aquaculture production of China is the largest in the world for many years with total annual production of 127.788 tons in 2008 [3]. In addition, the GFP aquaculture industry has also increased jobs and provides food for people. However, GFP aquaculture industry of China faces many problems such as disease, pollution, and undeveloped technology. Consequently, cultured GFP grow slowly in some areas, the time for one crop is prolonged, and the harvest size varies greatly, which has reduced the benefit to farmers recently.

Biotechnology has been developing rapidly and many researchers have applied this new technology to study diseases and their treatments, nutrition, environment interactions and genetic diversity of aquaculture species, including GFP. Although GFP is an important species for aquaculture, no complete genome of GFP in China exists to support scientists who research GFP growth in culture, disease prevention, nutrition, conservation, and other fields. Identifying the genes that affect phenotypic variation and important production traits is a very difficult and challenging task especially when only limited deoxyribonucleic acid (DNA) sequence information is published. A lack of basic information about the genome of GFP can be a major obstacle when developing improved aquaculture practices for production industries.

Techniques for molecular research have changed dramatically in recent years and the Next Generation Sequencing (NGS) technologies are currently the most important method for investigating the genomes of organisms, including aquatic animals. In general, NGS can be separated into three methods: 1) the Roche (454) Genome Sequencer FLX System based on sequencing-by-synthesis (pyrosequecing) technology was developed by 454 Life Sciences, as the first NGS platform available on the market [11]; 2) the Illumina (Solexa) Genome Analyzer sequencing platform was commercialized in 2006, based also on the principle of sequencing-by-synthesis chemistry; and 3) the Applied Biosystems SOLiD System, which is based on a sequencing-by-ligation technology. This platform has its origins in the system described by Shendure *et al.* (2005) [12] and in work by McKernan *et al.* (2006) [13] at Agencourt Personal Genomics (acquired by Applied Biosystems in 2006).

Some research on the genome of GFP has already been done. Jung *et al.* (2011) [14] used Roche 454 Genome Sequencing FLX technology to characterize the transcriptome of GFP using cDNA prepared from mRNA isolated from muscle, ovary and testes tissue. Maizatul *et al.* (2013) [15] used transcriptome sequencing of three tissue types: hepatopancreas, gill and muscle to generate functional genomics data for GFP at a massive scale.

In this study we collected GFP samples in Zhejiang province of China, because Zhejiang is a major production area of cultured GFP. Among the sequencing technologies, Illumina HiSeq 2500 has become the production platform of choice for all major genome centers and institutes around the world [16] because it has the biggest output, lowest reagent cost. We used this technology for de novo transcriptome sequencing analysis in order to provide functional annotation and classification and measure gene expression differences among four groups of GFP. This genetic information will support further research into this important species.

## Materials and Methods

### 2.1. Specimens

The GFP samples of this study were collected from two private prawn farms in Haiyan County, Zhejiang Province, China (This study did not involve in endangered or protected species; and no specific permissions were required for the animal experiments). Samples from each farm were further separated into two groups based on sizes, one was small and the other was big. That is, from Farm 1, we got samples of A1 and A2 groups (Owner of the farm 1: Mr. Guanxiang Ye; address: New village, Wuyuan Street, Haiyan county, Zhejiang province, China. Position: 30°30′25.87″ N; 120°54′50.30″E) and from Farm 2, we got samples of B1 and B2 groups (Owner of the farm 2: Mr. Sannan Bao, address: Daliu village, Wuyuan Street, Haiyan County, Zhejiang Province, China. Position: 30°32′32.54″ N; 120°54′28.49″E); the owner had agreed to let us collect the prawn samples for experiment. The GFP have been cultured for the same period of time and we extracted hepatopancreas from live GFP. Hepatopancreas is the major organ for metabolism and plays a vital role in the synthesis of digestive enzymes, and in secretion, nutrient absorption, digestion, excretion, reserve storage, mobilization [17], [18] and it is considered to be an important organ affecting crustacean growth [19]–[22]. At the first location sampled, we collected 24 samples for small size from 12 individuals (two samples of hepatopancreas per individual) and each sample was marked A1.1 to A1.24. The small GFP were measured with mean of length 68.75±5.85 cm and weight of 3.9±1.07 g. From the big GFP we collected 20 samples from 10 individuals and each sample was marked A2.1 to A2.20. The big GFP sampled had a mean of length 116.05±12.14 cm and weight of 20.82±6.92 g. At the second location we collected 24 samples for small size from 12 individuals and each sample was marked B1.1 to B1.24. These individuals had a mean length of 75.40±8.94 cm and weight of 4.60±1.47 g. At this location we collected 20 samples from 10 big GFP individuals and marked the samples B2.1 to B2.20. These individuals had a mean length of 125.10±12.57 cm and weight of 26.60±6.76 g (Table 1). All samples were immediately frozen in liquid nitrogen and stored at −70°C, thereafter they were transported to Shanghai Ocean University and put in to cold storage (–80°C).

**Table 1:** The detailed information on all sampled individuals used in this analysis.

No	Length (mm)	Weight (gr)	No	Length (mm)	Weight (gr)	No	Length (mm)	Weight (gr)	No	Length (mm)	Weight (gr)
**A1.1**	73.66	5.7	A2.1	137.02	37.6	B1.1	81.62	5.6	B2.1	136.45	37.8
**A1.2**			A2.2			B1.2			B2.2		
**A1.3**	67.85	5.1	A2.3	127.24	24.3	B1.3	61.52	2.1	B2.3	131.09	35.3
**A1.4**			A2.4			B1.4			B2.4		
**A1.5**	61.29	2.6	A2.5	140.15	34.7	B1.5	61.96	2.1	B2.5	141.2	33.8
**A1.6**			A2.6			B1.6			B2.6		
**A1.7**	54.15	2.1	A2.7	120.5	21.3	B1.7	83.75	5.4	B2.7	104.01	17.9
**A1.8**			A2.8			B1.8			B2.8		
**A1.9**	69.8	3.4	A2.9	110.4	15	B1.9	84.41	5.7	B2.9	107.17	20
**A1.10**			A2.10			B1.10			B2.10		
**A1.11**	73.25	4.2	A2.11	107.15	20.7	B1.11	82.5	6.1	B2.11	115.43	20.04
**A1.12**			A2.12			B1.12			B2.12		
**A1.13**	77.8	5.1	A2.13	110.96	14.5	B1.13	94.58	8.8	B2.13	131.93	22.2
**A1.14**			A2.14			B1.14			B2.14		
**A1.15**	74.49	4.5	A2.15	101.72	13.1	B1.15	79.39	4.7	B2.15	131.02	27.8
**A1.16**			A2.16			B1.16			B2.16		
**A1.17**	74.12	4.6	A2.17	100.49	12.1	B1.17	71.41	3.5	B2.17	111.02	19.3
**A1.18**			A2.18			B1.18			B2.18		
**A1.19**	61.09	2.2	A2.19	104.87	14.9	B1.19	75.2	4.4	B2.19	141.92	32.3
**A1.20**			A2.20			B1.20			B2.20		
**A1.21**	73.06	4.5				B1.21	68.5	3.5			
**A1.22**						B1.22					
**A1.23**	64.14	2.8				B1.23	60.34	3.1			
**A1.24**						B1.24					
**Mean**	68.725±5.85	3.9±1.07	Mean	116.05±12.14	20.82±6.92	Mean	75.4±8.94	4.6±1.47	Mean	125.1±12.57	26.6±6.76

### 2.2. RNA extraction

Total ribonucleic acid (RNA) was extracted from the tissue using TRIzol Reagent according the manufacturer’s instructions (Invitrogen) and genomic DNA was removed using DNase I (TaKara). Then RNA quality was determined by 2100 Bioanalyser (Agilent) and quantified using the ND-2000 (NanoDrop Technologies). Only high-quality RNA sample (OD260/280 = 1.8∼2.2, OD260/230≥2.0, RIN≥6.5, 28 S:18 S≥1.0, >10 µg) was used to construct sequencing library.

### 2.3. Library preparation and Illumina Hiseq2500 sequencing

RNA-seq transcriptome library was prepared following TruSeqTM RNA sample preparation Kit from Illumina (San Diego, CA) using 5 µg of total RNA. Messenger RNA was isolated according to polyA selection method by oligo (dT) beads and then fragmented (100 bp to 400 bp) by fragmentation buffer. Next, double-stranded Complementary to RNA (cDNA) was synthesized using a SuperScript double-stranded cDNA synthesis kit (Invitrogen, CA) with random hexamer primers (Illumina). Then the synthesized cDNA was subjected to end-repair, phosphorylation and ‘A’ base addition according to Illumina’s library construction protocol. Libraries were size selected for cDNA target fragments of 200–300 bp on 2% Low Range Ultra Agarose followed by Polymerase chain reaction (PCR) amplified using Phusion DNA polymerase (NEB) for 15 PCR cycles. After quantification by TBS380, a paired-end RNA-seq sequencing library was sequenced with the Illumina HiSeq 2500 (2×100 bp read length) [23], [24].

### 2.4. De novo assembly and annotation

The raw paired-end reads were trimmed and quality controlled by SeqPrep (https://github.com/jstjohn/SeqPrep) and Sickle (https://github.com/najoshi/sickle) with default parameters. Then clean data from the samples (A1, A2, B1 and B2) were used to do RNA de novo assembly with Trinity (http://trinityrnaseq.sourceforge.net/) [25]. All the assembled transcripts were searched against the NCBI protein NR, STRING, Swissprot, COG database [26], [27] and KEGG [28] database and KO (KEGG Ortholog database [29], [30]) using BLASTX. This was done to identify the proteins that had the highest sequence similarity with the given transcripts to retrieve their function annotations and a typical cut-off E-value less than 1.0×10^−5^ was set [31]. BLAST2GO (http://www.blast2go.com/b2ghome) [32] program was used to get GO [33] annotations of unique assembled transcripts for describing biological processes, molecular functions and cellular components. Metabolic pathway analysis was performed using the KEGG (http://www.genome.jp/kegg/) [34].

### 2.5. Differential expression analysis and functional enrichment

To identify DEGs between four different samples, the expression level of each transcript was calculated according to the fragments per kilobase of exon per million mapped reads (FRKM) method. RNASeq by Expectation Maximization (RSEM) (http://deweylab.biostat.wisc.edu/rsem/) [35] was used to quantify gene and isoform abundances. The R statistical package software EdgeR (Empirical analysis of Digital Gene Expression in R, http://www.bioconductor.org/packages/2.12/bioc/html/edgeR.html) [36] was used for differential expression analysis. In addition, functional-enrichment analysis including GO and KEGG [37] were performed to identify which DEGs were significantly enriched in GO terms and metabolic pathways at Bonferroni-corrected P-value ≤0.05 compared with the whole-transcriptome background. GO functional enrichment and KEGG pathway analysis were carried out by Goatools (https://github.com/tanghaibao/Goatools) and KOBAS (http://kobas.cbi.pku.edu.cn/home.do) [38].

## Results

### 3.1. Illumina Sequencing and Sequence Assembly

**Figure 1:**
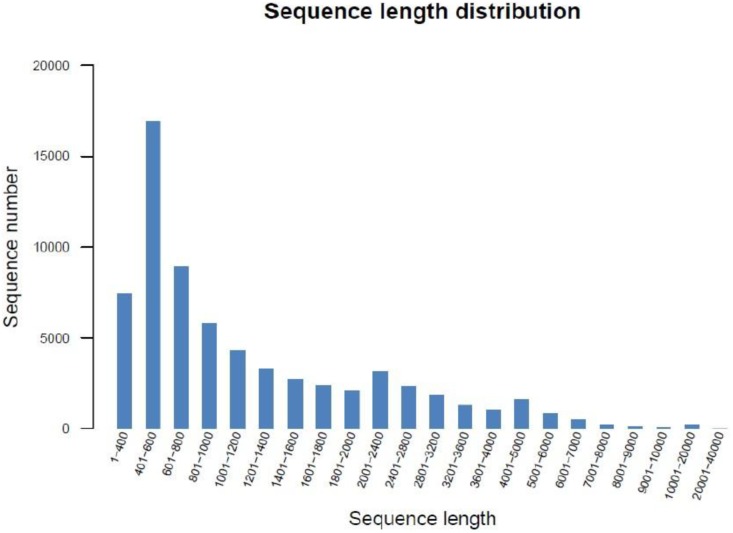
Assembed distribution of isogene lengths.

With the purpose of determining 4 groups of GFP (A1, A2, B1 and B2) transcriptomes, four sequencing libraries were prepared from the hepatopancreas of the GFP samples and sequenced with the Illumina paired-end technique (the Illumina HiSeq 2500 (2×100 bp read length)). In total, there were 95,133,630 raw reads generated from A1; 66,023,532 raw reads from A2; 63,157,060 raw reads from B1 and 59,321,096 raw reads from B2; the raw reads generated of all four groups A1, A2, B1 and B2 at Q20≥93.56%, 91.91%, 93.64% and 93.74%, respectively (Table 2). The sequencing raw data have been submitted to the SeqPrep (https://github.com/jstjohn/SeqPrep) and Sickle (https://github.com/najoshi/sickle) with default parameters. These were used for de novo assembly and resulting sequences of A1, A2, B1 and B2 groups were 89,527,020 clean reads; 58,980,778 clean reads; 59,479,630 clean reads and 55,842,210 clean reads, respectively (All sequences are being submitted to the NCBI Sequence Read Database). Using Trinity to process the transcription of assembled sequence identified a total 44,751 genes and 66,953 total isogenes. The lengths of all isogenes were distributed as 1,361.29 bp, 30,832 bp and 351 bp from average, largest and smallest length, respectively (Table 3). Of these, 16,943 (25.31%) were 401–600 bp; 8,912 (13.31%) were 601–800 bp; 7,423 (11.09%) were 1–400 bp; 5,757 (8.60%) were 801–1000 bp; 4,283 (6.40%) were 1001–1200 bp; 3,294 (4.92%) were 1,201–1,400 bp; 18,340 (27.39%) were 1,401–5,000 and 2,001 (2.99%) were 5,001–40,000 (Table 4, Figure 1) [25].

**Table 2:** Statistics of the sequencing results from all samples.

The sample name	Raw reads	Raw bases (bp)	≥ Q20 (%)	Clean reads	Clean bases (bp)	≥ Q20 (%)
**A1**	95 133 630	9 608 496 630	93.56	89 527 020	8 685 431 397	98.29
**A2**	66 023 532	6 668 376 732	91.91	58 980 778	5 712 404 876	98.07
**B1**	63 157 060	6 378 863 060	93.64	59 479 630	5 773 204 740	98.29
**B2**	59 321 096	5 991 430 696	93.74	55 842 210	5 427 406 640	98.33

Q20: The percentage of bases with a Phred value >20.

Note: The sequence length is 2 * 101 bp, that is, each read length of 101 bp, double-end sequencing.

**Table 3:** The assembled results.

Type	Description	Quantity
**Total genes**	Assembly number of genes	44 751
**Total isogenes**	The transcription of assembled	66 953
**Total residues**	Assembled from all of the isogenes (bp)	91 142 396
**Average length**	Assembled from transcript average length (bp)	1 361.29
**Largest isogene**	Assembled from the transcription of the longest length (bp)	30 832
**Smallest isogene**	Assembled from the transcription of the shortest length (bp)	351

**Table 4:** Assembled length distribution statistics for isogenes.

Isogene length (bp)	Quantity of Isogene	Percentage (%)
1–400	7,423	11.09%
401–600	16,943	25.31%
601–800	8,912	13.31%
801–1000	5,757	8.60%
1001–1200	4,283	6.40%
1201–1400	3,294	4.92%
1401–1600	2,725	4.07%
1601–1800	2,356	3.52%
1801–2000	2,116	3.16%
2001–2400	3,115	4.65%
2401–2800	2,307	3.45%
2801–3200	1,835	2.74%
3201–3600	1,275	1.90%
3601–4000	1,000	1.49%
4001–5000	1,611	2.41%
5001–40000	2,001	2.99%
**ALL**	**66,953**	**100.00%**

### 3.2. Functional annotation and classification

**Figure 2:**
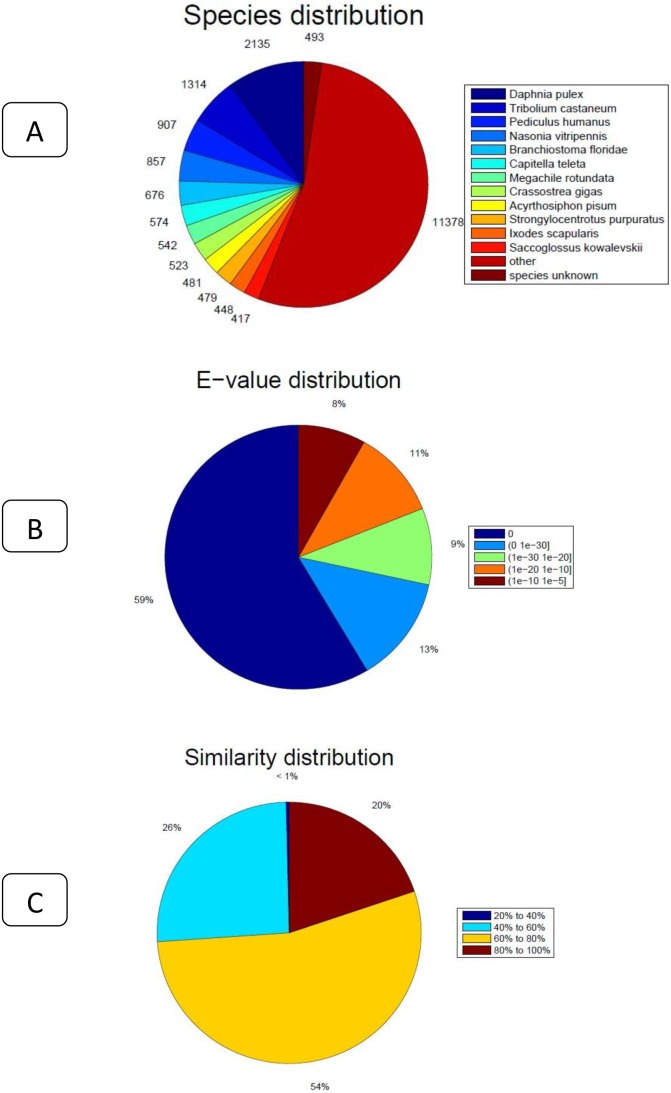
The distribution of Non-Redundant library comparisons.

**Figure 3:**
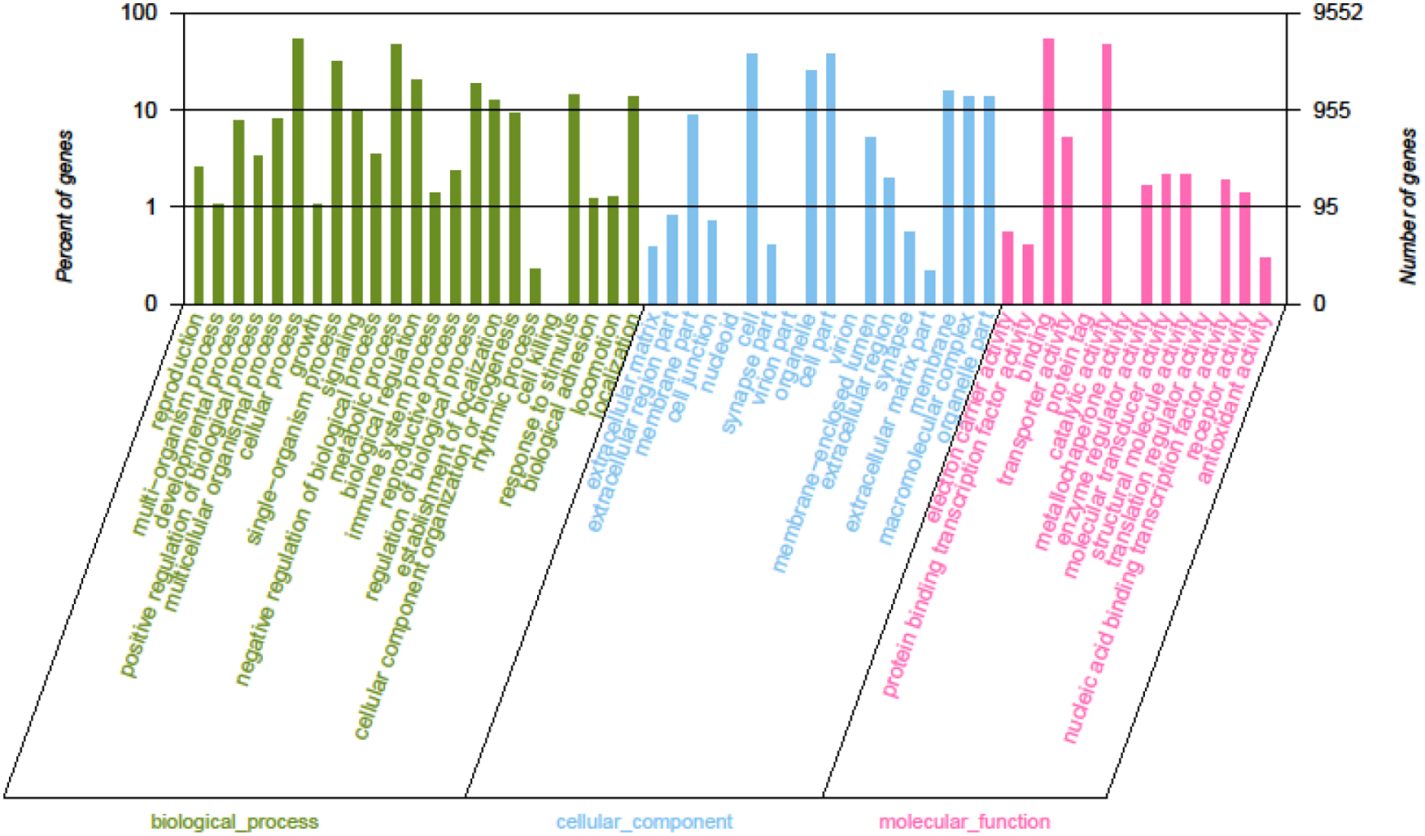
Gene Ontology classification of assembled isogenes. The 21,716 matched unigenes were classified into 3 functional categories: molecular_function, biological_process and cellular_component.

**Figure 4:**
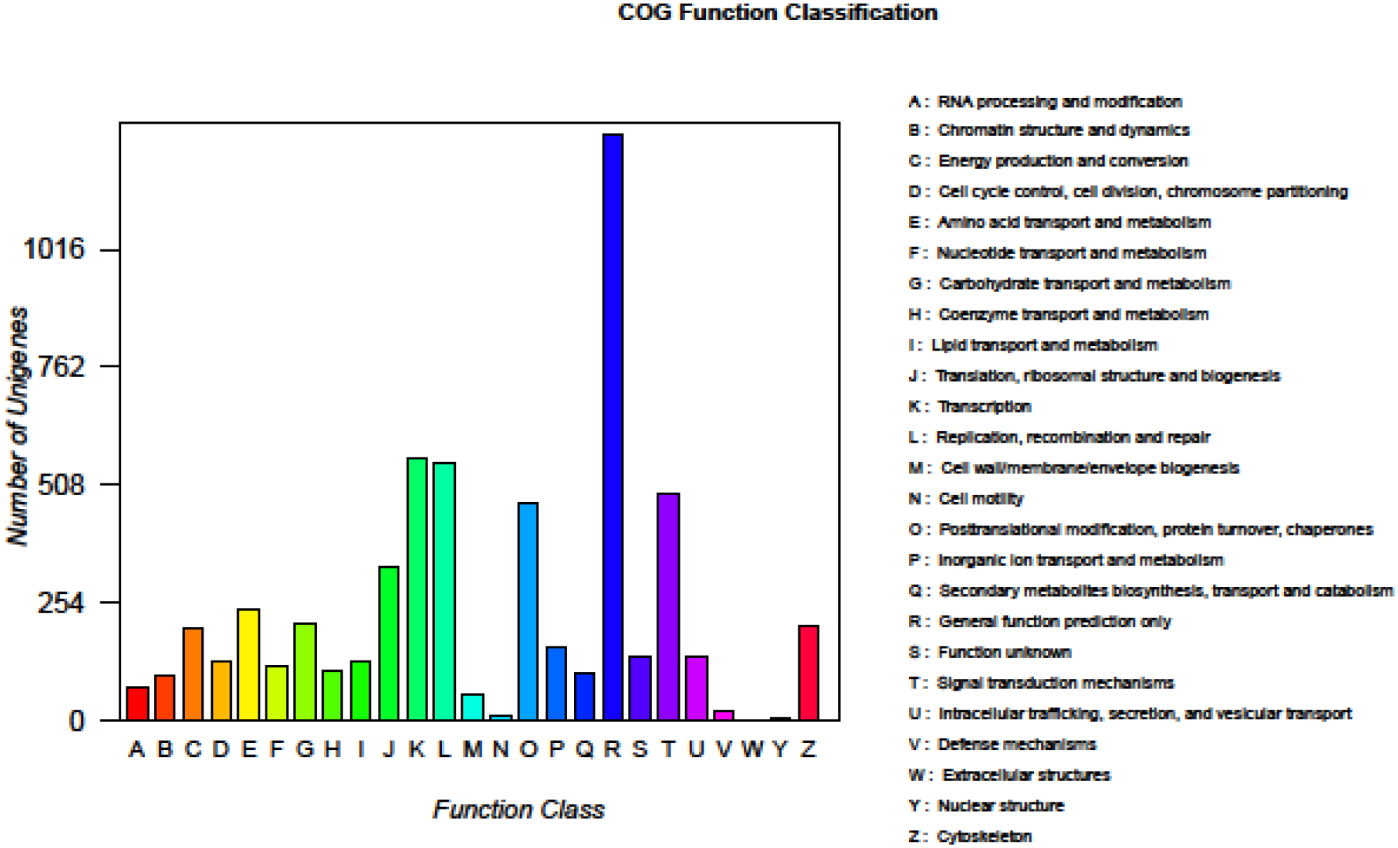
Clusters of Orthologous Groups of proteins functional classification of all isogenes sequences.

**Figure 5:**
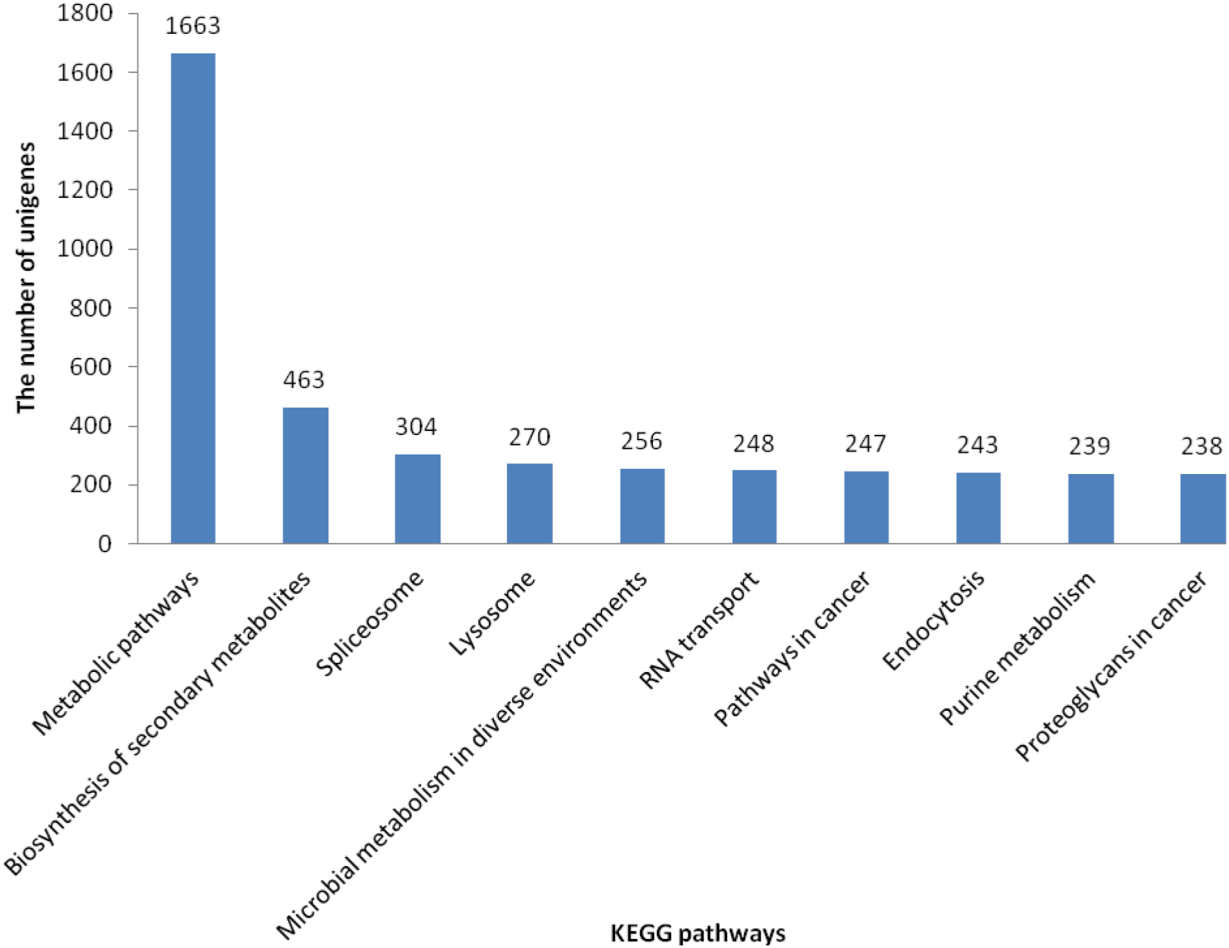
KEGG classification of isogenes: 20,859 unigenes were assigned into 312 KEGG pathways. The top 10 most abundant KEGG pathways are shown.

All the 66,953 assembled isogenes were searched against the NR, STRING, and KEGG databases using BLASTX (E-values less than 1.0×10^−5^ were set). Using BLAST2GO for GO annotation and KEGG for metabolic pathway analysis, a total of 21,224 unigenes was annotated, accounting for 31.7% (Figure 2A). The distribution of NR E-values was 59%, 13%, 9%, 11% and 8% proportional to E value 0, (0–1e^−30^), (1e^−30^–1e^−20^), (1e^−20^–1e^−10^) and (1e^−10^–1e^−5^), respectively (Figure 2B). In all unigene annotations, 20% showed high homology (80–100%) with sequences in the NR database. Of the remaining, 54% showed homology at 60–80%, 26% showed homology at 40–60%, and less than 1% showed homology at 20–40% with sequences in the NR database (Figure 2C) (Table S1). With the GO classification, the 9,552 matched unigenes (14.27% in total isogenes) were classified into 3 functional categories: molecular function, biological process and cellular component (Figure 3). In the category of molecular function, these matched unique sequences were clustered into 11 classifications. The largest subcategory of the molecular function was ‘binding’ (53.29%) and the second was ‘catalytic activity’ (47.56%). In the category of biological processes, these unique sequences were grouped into 22 classifications: the most represented biological processes were ‘cellular process’ (52.51%) and ‘metabolic process’ (46.38%). In the category of cellular components, these unique sequences were divided into 15 classifications: the most represented cellular components were ‘cell’ (37.9%) and ‘cell part’ (37.89%). The 5,782 matched unigenes (8.64% of the isogenes) were clustered into 25 categories of COG (Figure 4). The largest category was ‘general function prediction only’ (21.9%); the second category was ‘transcription’ (9.75%); the third category was ‘replication, recombination and repair’ (9.63%). When the assembled isogenes were assigned to the biochemical pathways described in KEGG, pathway-based analysis provided further understanding of the biological functions of the genes identified [39]–[41]. To systematically analyze inner-cell metabolic pathways and complicated biological behaviors, we classified the unigenes into biological pathways by mapping the annotated coding region sequences to the reference canonical pathways in the KEGG database (Figure 5). Consequently, 20,859 unigenes (31.15% of isogenes) were assigned to 312 KEGG pathways. Among these, 1,663 unigenes assigned to ‘metabolic pathways’, followed by ‘Biosynthesis of secondary metabolites’ (463 unigenes), ‘Spliceosome’ (304 unigenes), ‘Lysosome’ (270 unigenes), ‘Microbial metabolism in diverse environments’ (256 unigenes), ‘RNA transport’ (248 unigenes), ‘Pathways in cancer’ (247 unigenes), ‘Endocytosis’ (243 unigenes), ‘Purine metabolism’ (239 unigenes, ‘Proteoglycans in cancer’ (238 unigenes) (Table S2) [25], [42], [43].

### 3.3. Gene expression differences among four groups of GFP

**Figure 6:**
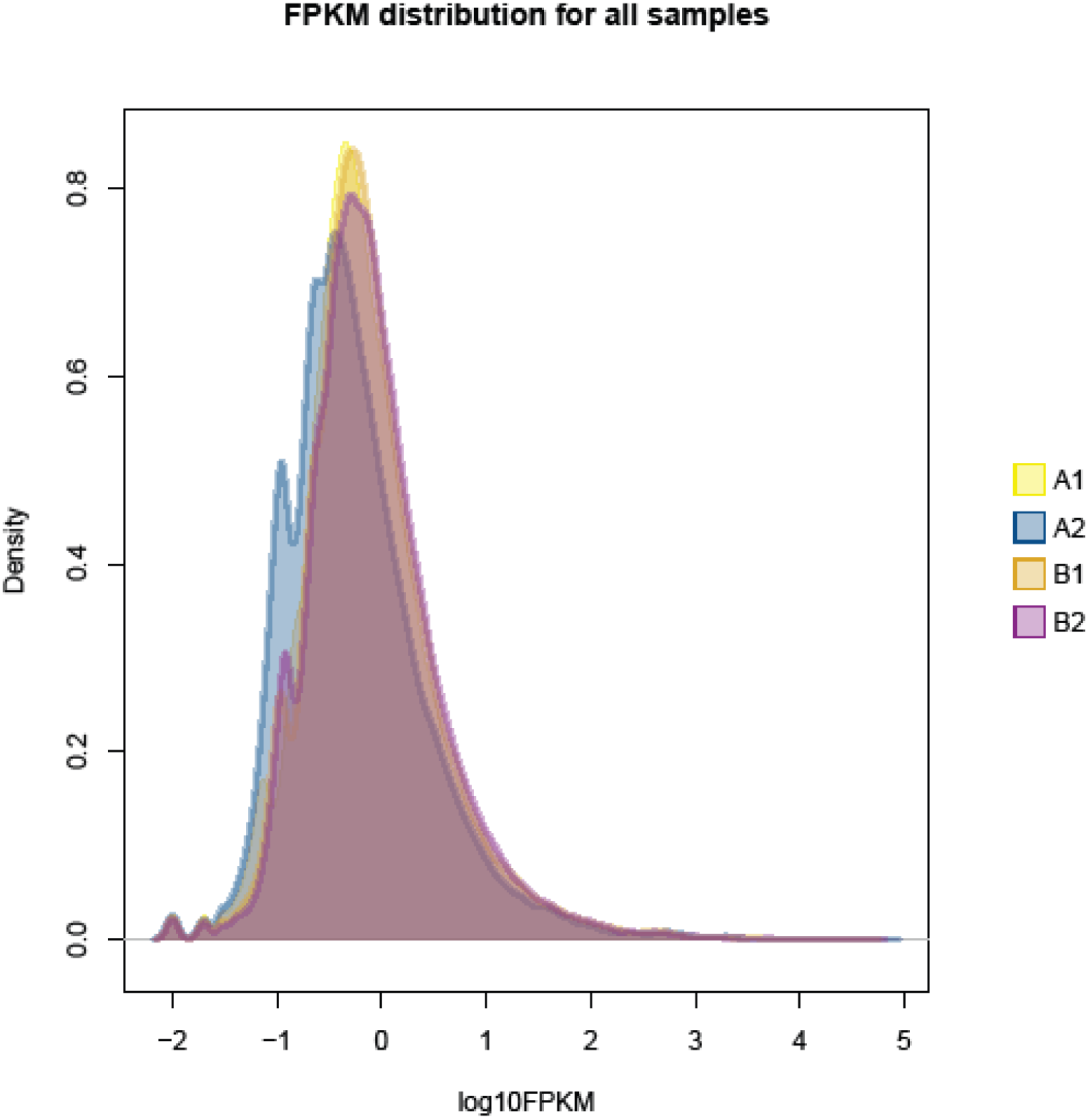
Express (FPKM scores) distribution. Left - all the probability density distribution of gene expression, the diagram for log10 FPKM abscissa, the higher the numerical, said the higher amount of gene expression.

Gene expression was calculated in accordance with the method of FPKM, which takes into account the influence of both the sequencing depth and gene length on read count. In the FPKM distribution for all samples (Figure 6) the A1 group showed the highest probability density distribution of gene expression, whereas A2 group displayed the lowest gene expression of probability density distribution. Among four GFP groups in this study, we used two types of genes for expression differences. Based on assembled total genes (44,751 genes) (Table 5). The results illustrated that 1,208 genes (2.7% of all genes) the A1 and A2 groups were identified as significant DEGs between these two groups (A1 and A2), which comprised 512 up-regulated genes (accounting for 42.38% of all significant DEGs) and 696 down-regulated genes (accounting for 57.62%) in the A1 group (Pearson correlation: 0.9379). Between the B1 and B2 groups, 1,668 genes (3.73% of all genes) were identified as significant DEGs, which comprised 1,518 up-regulated genes (accounting for 91.01% of all significant DEGs) and 150 down-regulated genes (accounting for 8.99%) in the B1 group (Pearson correlation: 0.9478). Between the A and B groups, 118 genes (1.53% of all genes) were identified as significant DEGs, which comprised 62 up-regulated genes (accounting for 52.54% of all significant DEGs) and 56 down-regulated genes (accounting for 47.46%) in the A group (Pearson correlation: 0.9834).

**Table 5:** The gene expression differences among four groups of GFP.

	Total genes			Total isogenes		
	Up-regulatedgenes	Down-regulatedgenes	Not differentiallyexpressed	Up-regulatedgenes	Down-regulatedgenes	Not differentiallyexpressed
**B/A**	62	56	13,610	91	56	16,583
**A2/A1**	512	696	43,543	2,421	4,439	60,093
**B2/B1**	1,518	150	43,083	3,586	1,643	61,724

**Figure 7:**
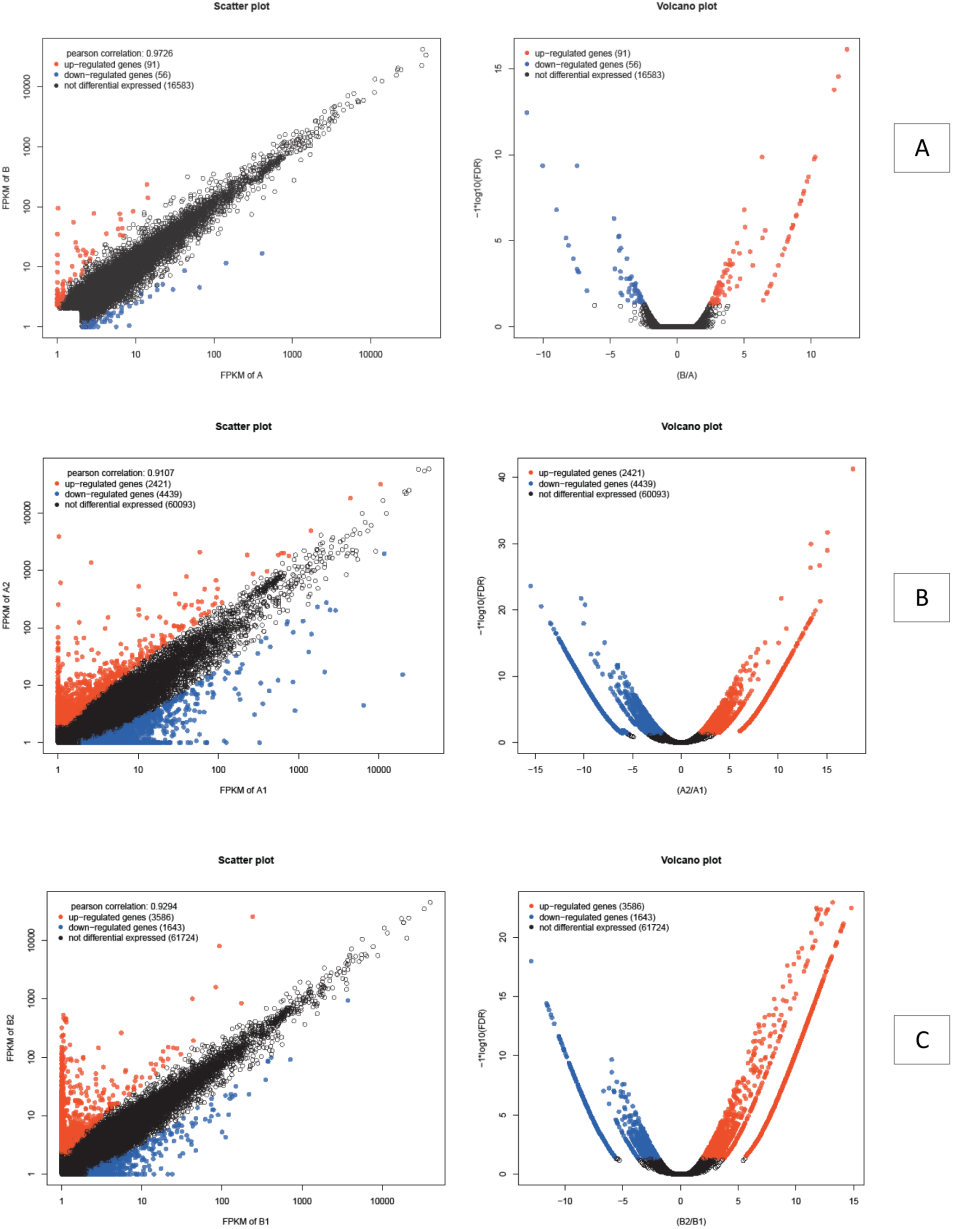
Visualization of differentially expressed gene transcription (scatter plot and volcanic plot) between A and B groups (A); A1 and A2 groups (B); B1 and B2 groups (C).

Based on assembled total isogenes (Table 5), 147 genes (0.22% of all isogenes) in the A and B groups were identified as significant DEGs between these two groups (A and B), which comprised 91 up-regulated genes (accounting for 61.9% of all significant DEGs) and 56 down-regulated genes (accounting for 38.1%) in the A group (Pearson correlation: 0.9704) (Figure 7A) (Table S3). Between the A1 and A2 groups, 6,860 genes (10.25% of all isogenes) were identified as significant DEGs, which comprised 2,421 up-regulated genes (accounting for 35.29% of all significant DEGs) and 4,439 down-regulated genes (accounting for 64.71%) in the A1 group (Pearson correlation: 0.9107) (Figure 7B) (Table S4). Between the B1 and B2 groups, 5,229 genes (7.81% of all isogenes) were identified as significant DEGs, which comprised 3,586 up-regulated genes (accounting for 68.23% of all significant DEGs) and 1,643 down-regulated genes (accounting for 31.77%) in the B1 group (Pearson correlation: 0.9294) (Figure 7C) (Table S5) [44]–[48].

### 3.4. Functional annotation of DEGs

**Figure 8:**
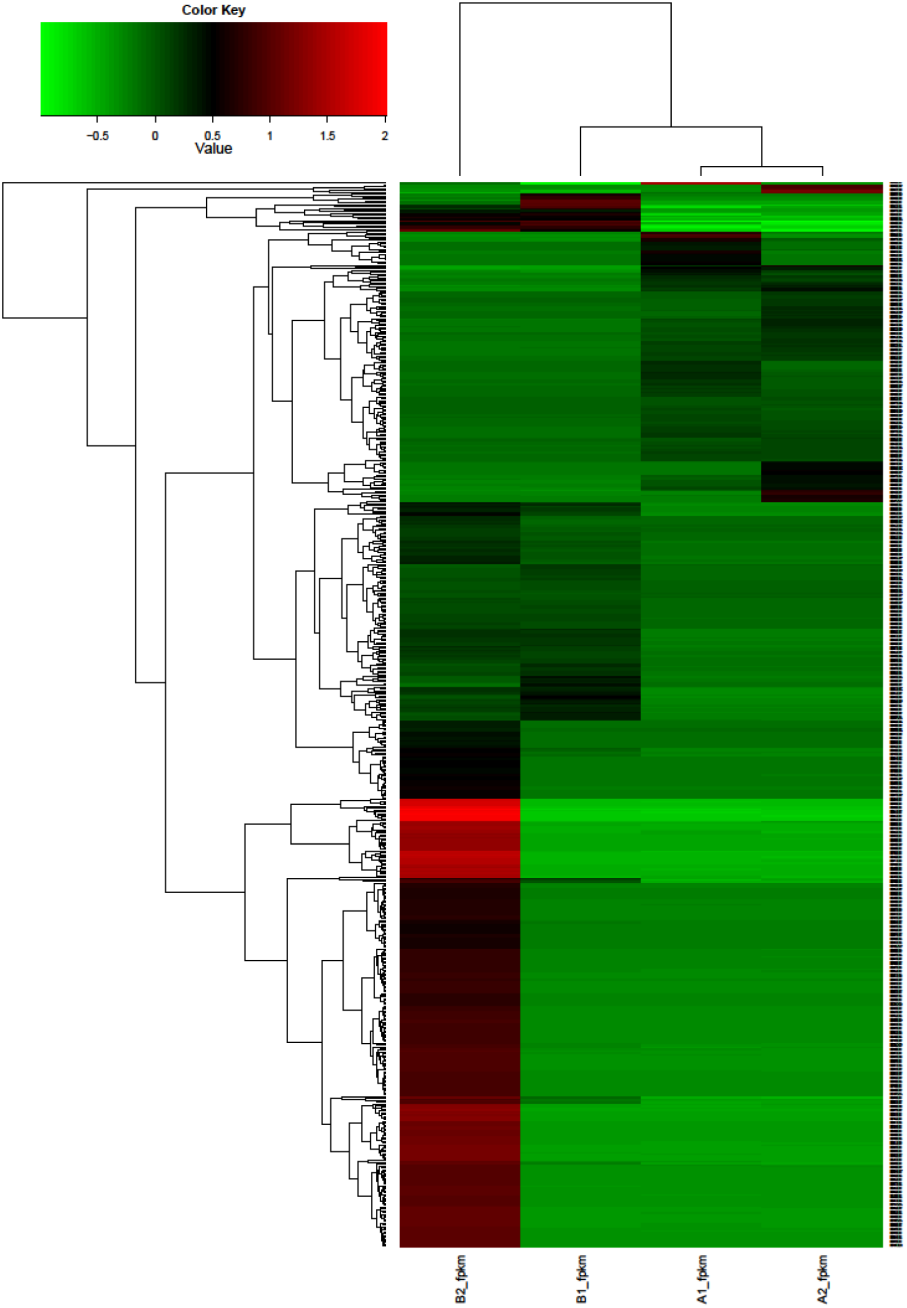
Hierarchical cluster analysis of common DEGs among 4 GFP groups.

A total of 147 DEGs between A and B groups, 6,860 DEGs between A1 and A2 and 5,229 DEGs between B1 and B2 were classified using hierarchical clustering (Figure 8) which indicated that A1 group was grouped closely with A2 group and that the B2 group was the most distinct of the set. The up-regulated and down-regulated genes were separated into two clusters based on tree branching.

**Figure 9:**
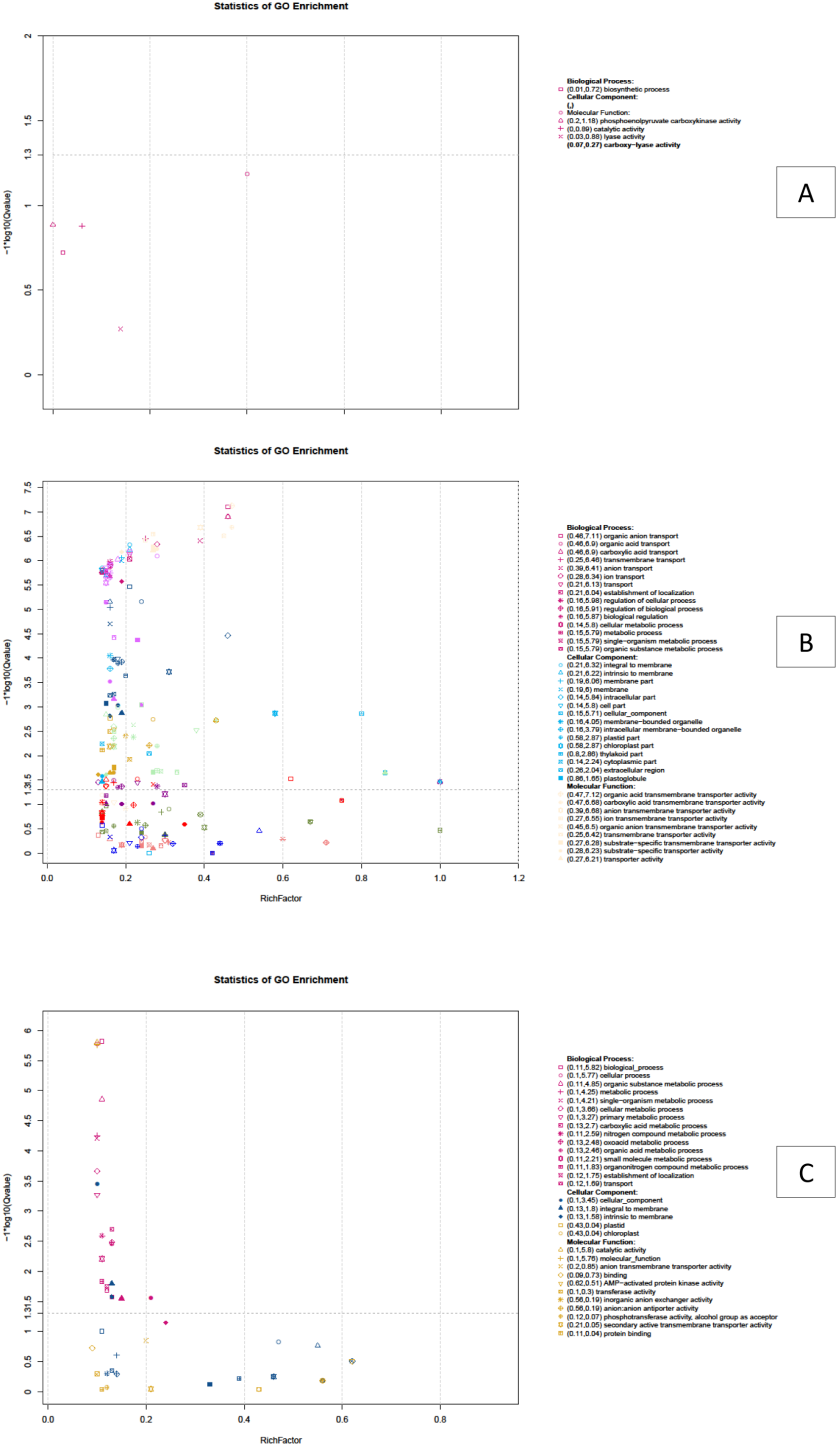
Scatterplot of differentially expressed genes from GO enrichment analysis between A and B groups (A); A1 and A2 groups (B); B1 and B2 groups (C).

All DEGs of A and B groups were mapped in the GO database to determine functions, looking for significantly enriched GO terms compared to the whole transcriptome background (total of isogenes) (Figure 9A). Using 147 DEGs between the A and B groups for enrichment we identified 38 DEGs that could be categorized into two main categories based on functional groups: biological process and molecular function. In biological_process the predominant term was ‘biosynthetic process’ with 8 DEGs (corrected P value = 0.19). In molecular_function four predominant terms were found, those were ‘phosphoenolpyruvate carboxykinase activity’ (2 DEGs, corrected P value = 0.0654), ‘catalytic activity’ (22 DEGs, corrected P value = 0.13), ‘lyase activity’ (4 DEGs, corrected P value = 0.132), and ‘carboxy-lyase activity’ (2 DEGs, corrected P value  = 0.535) (Table S6). Using 6,860 DEGs between A1 and A2 groups for enrichment, we derived 21,856 DEGs (corrected P value≤0.05), because some genes occurred in more than one term (Figure 9B). These DEGs could be categorized into three main categories based on functional groups: biological process had 10,013 DEGs in 68 terms, of which the 30 predominant terms have more than 100 DEGs each. The highest was the ‘biological_process’ term with 1,005 DEGs. Molecular_function had 8,572 DEGs in 55 terms, of which the 23 predominant terms have more than 100 DEGs. The highest was the ‘molecular_function’ term with 1,275 DEGs. Cellular_component had 3,271 DEGs in 19 terms, of which the 13 predominant terms had more than 100 DEGs. The highest was ‘Cellular_component’ term with 687 DEGs (Table S7). Using 5,229 DEGs between B1 and B2 groups for enrichment, we derived 5,940 DEGs (corrected P value≤0.05) (Figure 9C), some genes occurred in more than one term. These DEGs could be categorized into three main categories based on functional groups: biological process had 4,077 DEGs in 18 terms, most of which have more than 100 DEGs; the highest was ‘biological_process’ term with 656 DEGs. Molecular_function had 1,275 DEGs in 2 terms; one was ‘catalytic activity’ term with 475 DEGs and other was ‘molecular_function’ with 800 DEGs. Cellular_component had 588 DEGs in 3 terms including ‘cellular_component’ with 444 DEGs, ‘integral to membrane’ with 72 DEGs and ‘intrinsic to membrane’ with 72 DEGs (Table S8) [49]–[53].

**Figure 10:**
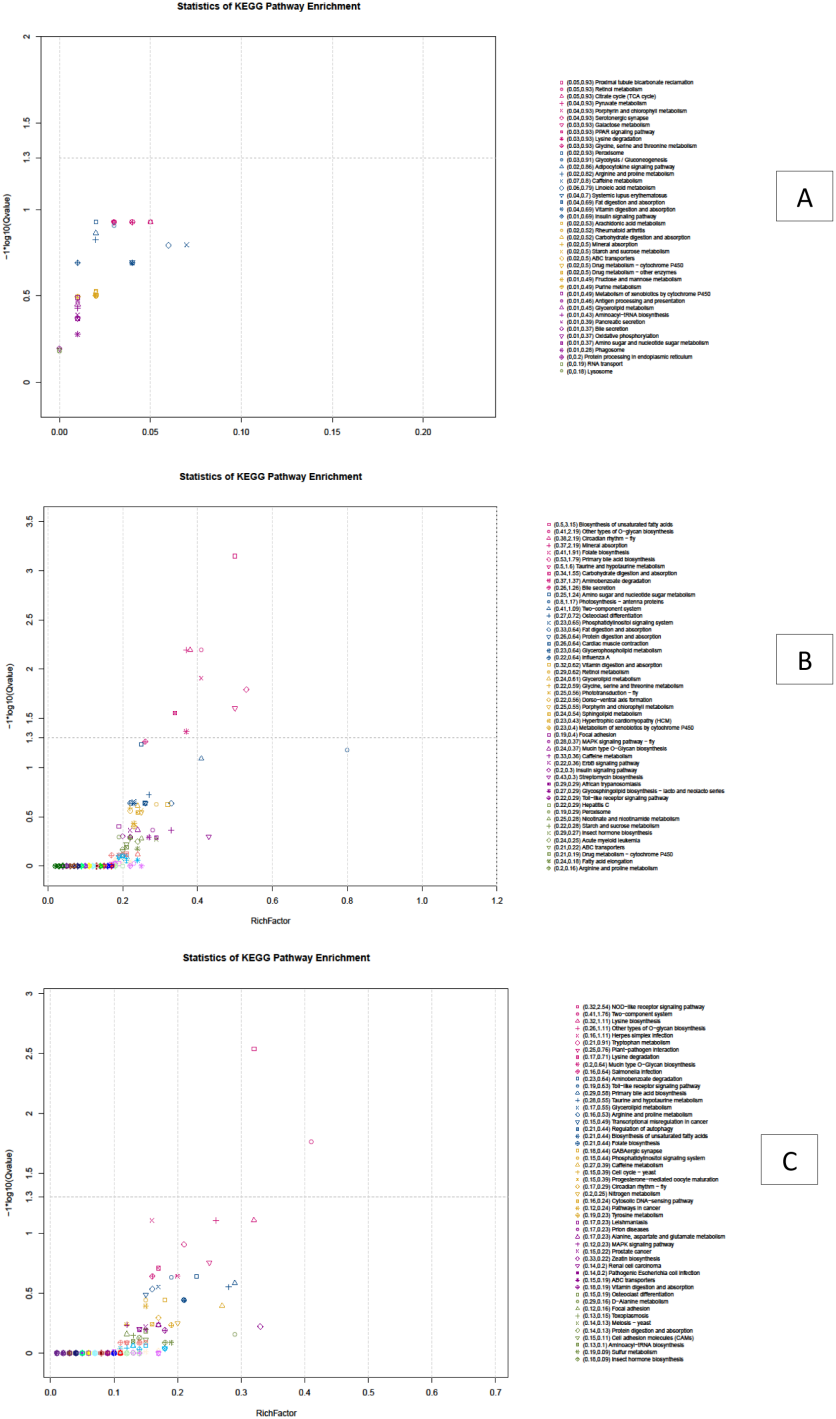
Scatterplot of differentially expressed genes from KEGG PATHWAY enrichment analysis between A and B groups (A); A1 and A2 groups (B); B1 and B2 groups (C).

To further investigate the biochemical pathways of these DEGs, all DEGs were mapped in the KEGG database and compared to the gene sequencing of background (isogenes) (Figure 10A). Of the 147 DEGs between A and B groups, 61 DEGs had a KO-ID and could be categorized into 42 pathways. None of the DEGs were significantly enriched (corrected P value≤0.05). Using 6,860 DEGs between A1 and A2 groups to investigate biochemical pathways, 2,459 DEGs had a KO-ID and could be categorized into 251 pathways (Figure 10B); of those, 9 pathways were significantly enriched at corrected P value≤0.05 and 4 pathways were significantly enriched at corrected P value≤0.01, those are ‘Biosynthesis of unsaturated fatty acids’, ‘Mineral absorption’, ‘Other types of O-glycan biosynthesis’ and ‘Circadian rhythm-fly’. Using 5,229 DEGs between B1 and B2 to investigate biochemical pathways, 1,543 DEGs had a KO-ID and could be categorized into 240 pathways (Figure 10C); of those 2 significantly enriched pathways (corrected P value≤0.05) were ‘NOD-like receptor signaling pathway’ (corrected P value = 0.002899) and ‘Two-component system’ (corrected P value = 0.0173) [38], [49].

### 3.5. The DEGs of interest for GFP growth among 4 groups

**Figure 11:**
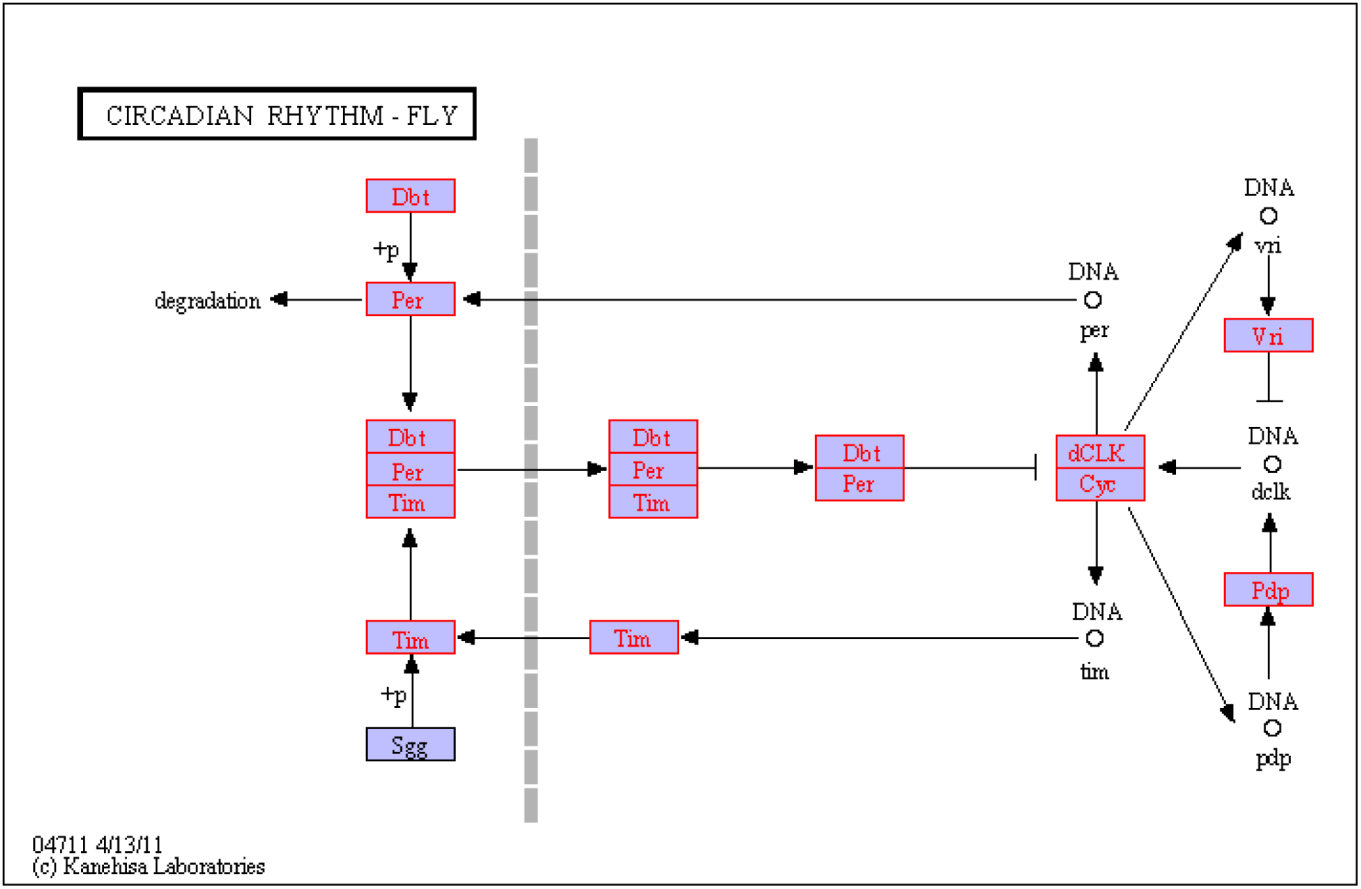
The KEGG PATHWAY is ‘ko 04711’ with term ‘Circadian rhythm - fly’.

In the current study, we identified the 99 queries (genes) involving to growth of GFP (99 queries of 9,552 matched unigenes of GO) (Table S9); of those 59 queries were named clearly (gene-name) and 60 queries were deposited in NCBI (gene-id). The 40 queries remainder have nearly no information, those may be novel genes and which would be done for next study. The 33 queries were identified between the A and B groups of 99 queries involving to growth of GFP, but none of the DEGs were significantly enriched (corrected P value≤0.05). The 67 queries were identified between A1 and A2 groups of 99 queries involving to growth of GFP and the 12 queries were significantly enriched (corrected P value≤0.05); of those, A2/A1 have 10 down-regulated genes and 2 up-regulated genes. In the 12 queries, 6 queries were defined gene-name and gene-id (gene-name: LGMN, HLF, ANK; gene-id: K01369, K09057, K10380); all of remainder have not defined gene-name and gene-id yet (Table 6). The 71 queries were identified between B1 and B2 groups of 99 queries involving to growth of GFP, and the 12 queries were significantly enriched (corrected P value<0.05), of those B2/B1 have 8 up-regulated genes and 4 down-regulated genes. In the 12 queries, just 3 queries were defined gene-name and gene-id (gene-name: CDC2L, IGF1R and PFN; gene-id: K08818, K05087 and K05759); all of remainder have not defined gene-name and gene-id yet (Table 6). We used the hypergeometric test method for testing the most enriched pathway. None of the KEGG PATHWAY were significantly enriched (corrected P value≤0.05) between A and B groups. The 9 KEGG PATHWAYS were significantly enriched (corrected P value≤0.05) between A1 and A2 groups, of those one KEGG PATHWAY is ‘ko04711’ with term ‘Circadian rhythm-fly’ has occurred 4 queries relating with GFP growth (comp63866_c0_seq9, comp63866_c0_seq14, comp63866_c0_seq10 and comp63866_c0_seq1) (Figure 11). The 2 KEGG PATHWAYS were significantly enriched (corrected P value≤0.05) between B1 and B2 groups (ko04621 and ko02020), but none of the KEGG PATHWAYS have occurred the queries relating with GFP growth.

**Table 6:** The DEGs relationships with GFP growth after enrichment of four groups.

Query	Gene-id	Gene-name	Qeury_length (bp)	Up-down-regulation1(A2/A1)	Significant(FDR< = 0.05,|logFC|> = 1)
**The A1 and A2 groups**					
comp45171_c0_seq1	K01369	LGMN	509	down	yes
comp63866_c0_seq9	K09057	HLF	318	down	yes
comp63078_c0_seq9	None	_	559	down	yes
comp21721_c0_seq1	_	_	375	down	yes
comp46864_c0_seq1	K10380	ANK	773	down	yes
comp63866_c0_seq14	K09057	HLF	304	up	yes
comp63078_c0_seq1	None	_	572	down	yes
comp63078_c0_seq3	None	_	630	down	yes
comp49661_c0_seq2	_	_	733	up	yes
comp63866_c0_seq10	K09057	HLF	351	down	yes
comp63866_c0_seq1	K09057	HLF	351	down	no
comp63078_c0_seq6	None	_	563	down	yes
**The B1 and B2 groups**				**Up-down-regulation1(B2/B1)**	
comp65788_c0_seq2	_	_	1891	up	yes
comp65617_c0_seq2	None	_	524	up	yes
comp66054_c0_seq4	None	_	647	up	yes
comp66702_c0_seq5	None	_	165	up	yes
comp67315_c0_seq4	K08818	CDC2L	713	up	yes
comp65788_c0_seq3	_	_	1,846	down	yes
comp780278_c0_seq1	K05087	IGF1R	194	up	yes
comp66702_c0_seq7	None	_	165	up	yes
comp49661_c0_seq2	_	_	733	down	yes
comp48084_c0_seq1	K05759	PFN	127	up	yes
comp63078_c0_seq2	None	_	278	down	yes
comp66054_c0_seq5	None	_	532	down	yes

## Discussion

There have been some researches on the GFP and *Litopenaeus vannamei* using the next generation sequencing technologies by Jung *et al.* (2011), Maizatul *et al.* (2013), Suchonma *et al.* (2013), Keyi *et al.* (2012) [14], [15], [54], [55], but they used the different transcriptome method with different experiment objectives.

In the current study, the number of clean reads varied from 55,842,210 to 89,527,020 (>98%) among the 4 GFP groups and 66,953 isogenes were assemble with varying lengths from 1 to 40,000 bp and average of length was 1,361.29 bp. This is higher than reported in previous research because we used the newest NGS technology for experiment (Illumina Hiseq 2500) which can read longer sequences. There was a difference in the number of clean reads among 4 GFP groups in this study, which may be the result of differences among the original broodstock or a change of each group according to culture time.

The large number of raw read sequences and the high ratio of derived clean read sequences in this study when compared with previous studies may be the result of using different technology for sequencing or the result of the size of samples used in the study. We noted that only 21,224 (31.7%) unigenes matched the registered sequences of GFP in the GenBank NR database, and 45,729 isogenes were newly discovered in our present result. This result suggests that we have made a meaningful contribution to the knowledge of GFP by characterizing these unigenes. The inability to find matches may be due to the lack of genomic information in non-model species [55] or it is also possible that some may constitute novel genes unique to this species [56]–[58]. Similarly, GO classification matched only 14.27% in total of 66,953 isogenes, a lower ratio than the results by Suchonma *et al.* (2013) [54] (data not shown), but higher than results by Maizatul *et al.* (2013) [15] (7,533 protein annotated unigenes). This may be caused by different technologies applied in these studies. However, the low level at which proteins annotated was expected due to the limited number of GFP protein sequences currently available in the NCBI databases [14]. The proteins annotated were classified into 3 functional categories and 48 terms in this study; more than previously reported by Maizatul *et al.* (2013) [15] (42 terms).

The total number of GO terms obtained in our study was larger than the total number of the unigenes, because several of the sequences were assigned to more than one GO term. In summary, terms account for a large fraction of the overall assignments in the GFP transcriptomic dataset. Understandably, genes encoding these functions may be more conserved across different species, thus they will be easier to annotate in the database [55]. Metabolic pathways were associated with a large number of unigenes with KEGG classifications in our study. These pathways are implicated in the kinetic impairment of muscle glutamine homeostasis in adult and old glucocorticoid-treated rats [59]. The unigenes assigned to KEGG pathways was relatively high in our study compared with the results by Jung *et al.* (2011) [14] (data not shown) and by Maizatul *et al.* (2013) [15]. Although we could not report all of unigenes which assigned in putative KEGG pathways, this database may provide insight into the specific responses and functions involved in molecular processes in GFP metabolism. The hierarchical cluster (Figure 11) of 4 groups indicated that they have a close genetic relationship, especially between A1 and A2, The B1 group has relatively close genetic distance with A1 and A2 groups and B2 has furthest genetic distance with 3 groups remainder. This may be caused by sampling individuals from different broodstock.

The number of DEGs among the 4 groups was less than results by Maizatul *et al.* (2013) [15], because the samples in our study were extracted from hepatopancreas organ of GFP, while Maizatul *et al.* (2013) [15] used different tissues for their experiment (hepatopancreas, gill and muscles). However the differentiation of DEGs between three pairs (The A and B; A1 and A2; B1 and B2) was relatively clear. The ratio between DEGs and total isogenes of three pairs A/B, A1/A2 and B1/B2 were 0.89%, 10.25% and 7.81% respectively, compared with previous studies, those ratios were relatively high [60]. In this study we identified 99 queries (GO-id) relationship with GFP growth (Figure 4). Among four GFP groups, after enriched, there were defined 6 gene names and 6 gene ids (9 queries of 23 queries) involving to growth of GFP.

The gene-id is K01369 (gene name: LGMN) with String_tophit_description is ‘hypothetical protein’. This gene was being definition ‘legumain’ [61]. Legumain is an enzyme [62]–[64], it catalyses the following chemical reaction for hydrolysis of proteins and small molecule substrates at -Asn-Xaa- bonds. This enzyme is present in legume seeds, the trematode Schistosoma mansoni and mammalian lysosomes. The gene-id is K09057 (gene name: HLF) with String_tophit_description is ‘par domain protein [Culex par domain protein [Culex quinquefasciatus]’, this gene was being definition ‘hepatic leukemia factor’. ‘Hepatic leukemia factor’ is a protein that in humans is encoded by the HLF gene [65]. It is suggesting that HLF plays a role in the function of adult differentiated neurons. This gene encodes a member of the proline and acidic-rich protein family, a subset of the Basic Leucine Zipper (bZIP) transcription factors. The bZIP transcription factors are effectors downstream of mitogenic stimulation, stress responses, and cytokine stimulation. Additionally, the bZIP family of transcription factors affects several developmental processes including dendritic cell development, myeloid differentiation, and brain and ocular development. The bZIP transcription factors are found in all organisms. Interactions between bZIP transcription factors play important roles in cancer development [66] in epithelial tissues, steroid hormone synthesis by cells of endocrine tissues [67], factors affecting reproductive functions [68] and several other phenomena that affect human health. The gene-id is K10380 (gene name: ANK) with String_tophit_description is “PREDICTED: hypothetical protein LOC409051 [Apis mellifera]”. The ANK gene was being definition ‘ankyrin’; Ankyrins are a family of adaptor proteins that mediate the attachment of integral membrane proteins to the spectrin-actin based membrane cytoskeleton [69]. The cytoskeleton is present in all cells. It forms structures such as flagella, cilia and lamellipodia and plays important roles in both intracellular transport and cellular division. Cytoskeletal elements interact extensively and intimately with cellular membranes [70]. A number of small molecule cytoskeletal drugs have been discovered that interact with actin and microtubules [71]. The gene-id is K08818 (gene name: CDC2L) with String_tophit_description is ‘novel protein similar to cell division cycle 2-like family [Xenopus (Silurana) tropicalis]’, this gene was being definition ‘cell division cycle 2-like’ [72]. The CDC2L genes encode almost identical protein kinases, the PITSLRE kinases, which have functions that may be relevant to the regulation of transcription/splicing and apoptotic signaling. Cyclin-dependent kinases (CDKs) are a family of protein kinases first discovered for their role in regulating the cell cycle. They are also involved in regulating transcription, mRNA processing, and the differentiation of nerve cells [73]. CDKs phosphorylate their substrates on serines and threonines, so they are serine-threonine kinases [73]. Animal cells contain at least nine CDKs, four of which, Cdk1, 2, 3, and 4, are directly involved in cell cycle regulation (Morgan, David O, 2007). The gene-id is K05087 (gene name: IGF1R) with String_tophit_description is ‘insulin receptor, putative [Ixodes scapularis]’, this gene was being definition ‘insulin-like growth factor 1 receptor’ [74]. The insulin-like growth factor 1 (IGF-1) receptor is a protein found on the surface of human cells. It is a transmembrane receptor that is activated by a hormone called insulin-like growth factor 1 (IGF-1) and by a related hormone called IGF-2. IGF-1 plays an important role in growth and continues to have anabolic effects in adults - meaning that it can induce hypertrophy of skeletal muscle and other target tissues [75]. The gene-id is K05759 (gene name: PFN) with String_tophit_description is ‘profilin [Penaeus monodon]’ this gene was being definition ‘profilin’ [76]. Profilin is an actin-binding protein involved in the dynamic turnover and restructuring of the actin cytoskeleton. It was first described in the early 1970s as the first actin monomer binding protein [77]. Profilin is important for spatially and temporally controlled growth of actin microfilaments which is an essential process in cellular locomotion and cell shape changes. Profilin also binds sequences rich in the amino acid proline in diverse proteins. While most profilin in the cell is bound to actin and it seems to be involved in activities in the nucleus such as mRNA splicing [78]. The KEGG PATHWAY was named ‘ko04711’ which was described by Stanewsky R. (2003) [79]. Its name was ‘Circadian rhythm - fly’. Circadian rhythm is an internal biological clock, which enables to sustain an approximately 24-hour rhythm in the absence of environmental cues. The core of the circadian clock consists of molecular feedback loops where per and tim gene products (PER and TIM proteins) eventually turn off their own transcription. The large number of queries in this study that were not functionally annotated and classified may be the result of limited availability of genetic information in Genbank or they are novel genes of GFP.

In summary, in this study we identified many DEGs after enrichments among the 4 groups. However, no DEGs with relationships to GFP growth were identified between A and B groups which came from different places (corrected P value<0.05). But, between A1 and A2 and between B1 and B2 groups were identified 23 DEGs relationships with growth of GFP; of those have positive and negative impact to grow of GFP. The results showed 4 queries (between A1 and A2 groups) joined in KEGG PATHWAY with name ‘Circadian rhythm - fly’. All of significantly enriched of DEGs and KEGG PATHWAY (corrected P value≤0.05) in this study were derived as proof for separated of GFP in pond into big and small groups. Obviously, there is not only genetics impact to grow of GFP, but also many factors impact to grow of GFP in pond such as diseases, environment, nutrition, etc.

## Conclusions

In present study we used the Illumina HiSeq 2500 for de novo sequencing which is the newest technology in NGS. We also used the published dataset from GenBank and available resources for analyzing the prawn transcriptome data through the model-based methods GO and KEGG pathway. We identified 66,953 assembled isogenes and 44,715 genes but just 21,224 unigenes were matched and annotated. The large number of isogenes and genes that were not functionally annotated and classified may be the result of limited availability of genetic information in Genbank or they are novel genes of GFP. In this study we also obtained gene expression differences among four groups of GFP and functional annotation of DEGs based on enriched GO and KEGG PATHWAY. Especially, we identified 23 DEGs relation with GPF growth. The results provide good information for further research on the mechanisms of DEGs with freshwater prawn growth processes. The functional genomics resource generated from our study provides a resource for future molecular research in freshwater prawn.

## Supporting Information

Table S1 **The annotation and classification of isogenes.**
(XLS)

Table S2 **The KEGG Pathway_definition.**
(XLS)

Table S3 **The DEGs between the A and B groups.**
(XLS)

Table S4 **The DEGs between the A1 and A2 groups.**
(XLS)

Table S5 **The DEGs between the B1 and B2 groups.**
(XLS)

Table S6 **The results after enrichment of DEGs between A and B groups.**
(XLT)

Table S7 **The results after enrichment of DEGs between A1 and A2 groups.**
(XLS)

Table S8 **The results after enrichment of DEGs between B1 and B2 groups.**
(XLS)

Table S9 **The 99 queries (GO) relationships with GFP growth.**
(XLS)
